# Risk factors for tooth loss with a mean follow-up period of 13.9 years in supportive periodontal therapy patients

**DOI:** 10.1186/s12903-021-01573-5

**Published:** 2021-04-22

**Authors:** Tomotaka Kato, Natsuki Fujiwara, Tomohisa Ogawa, Yukihiro Numabe

**Affiliations:** 1 Division of General Dentistry, Nippon Dental University Hospital, 2-3-16 Fujimi Chiyoda-ku, Tokyo, Japan; 2 Department of Periodontology, The Nippon Dental University School of Life Dentistry at Tokyo, Tokyo, Japan; 3 Fujiwara Dental Clinic, Hiroshima, Japan

**Keywords:** Tooth loss, Non-vital tooth, Retrospective cohort study, Multiple classification analysis, Supportive periodontal therapy

## Abstract

**Background:**

Clinical evidence indicates that there are various risk factors of tooth loss. However, the degree of this risk among other risk factors remains unclear. In this retrospective cohort study, the authors evaluated the hazard ratios of several risk factors for tooth loss.

**Methods:**

Included patients had all been treated for dental disorders, were in the supportive phase of periodontal therapy by dental hygienists, and visited a Japanese dental office continually during a 10-year period. Periodontal parameters, tooth condition, and general status of all teeth (excluding third molars) at the initial visit and at least 10 years later were evaluated by using multiple classification analysis.

**Results:**

The authors evaluated a total of 7584 teeth in 297 patients (average age: 45.3, mean follow-up time: 13.9 years) Non-vital pulp was the most significant predictor of tooth loss according to Cox hazards regression analysis (hazard ratio: 3.31). The 10-year survival rate was approximately 90% for teeth with non-vital pulp and 99% for teeth with vital pulp. Fracture was the most common reason for tooth loss.

**Conclusions:**

Non-vital pulp had the most significant association with tooth loss among the parameters. Therefore, it is very important to minimize dental pulp extirpation.

## Introduction

In the past several years, the importance of oral health for general health and wellbeing has been recognized [1, 2]. Tooth loss is often used as an indicator of oral health and is also related to general health [3–5]. Therefore, it is very important to understand the risk factors for tooth loss.

Dental caries and periodontal disease are the most common indications for the extraction of permanent teeth [6]. Age and sex are also risk factors for tooth loss in observational studies [7, 8]. Additional risk factors for dental extraction include smoking, diabetes mellitus, and use of a dental prosthesis; molars are at higher risk of extraction than other teeth [9–12].

Many studies have found that dental caries is most strongly related to tooth loss [11, 13–16]. However, some reports have shown that periodontal disease causes a greater proportion of tooth extractions than other factors [17]. It remains unclear which factor influences tooth loss more: dental caries or periodontal disease. In the clinical setting, non-vital pulp is a well-known risk factor for tooth extraction [17]. In our previous study, we reported that non-vital teeth were related to periodontal disease [18]. In addition, non-vital pulp is a risk factor for dental caries [17]. Therefore, we hypothesized that non-vital pulp is also a risk factor for tooth loss. However, there are insufficient data available to conclude that non-vital pulp is clearly a risk factor for tooth loss [17]; it remains unclear which risk factors are most closely related to tooth loss. Therefore, the purpose of this study was to clarify the reasons for extraction of permanent teeth and to identify which of the following factors has the strongest relationship with extraction: patient age, sex, and smoking habits; presence of diabetes mellitus, dental caries, periodontal disease, dental prosthesis, and non-vital pulp; and tooth position (molar vs other).

## Methods

### Subjects

This retrospective cohort study was performed at a general dental office from 2001 to 2019. Patients aged 18 to 81 years who had been treated for periodontal disease and other dental disorders and had been under supportive periodontal therapy (SPT) afterwards. The study included all patients who visited the dental office continually over a 10-year period at least once a year. We excluded patients who did not want to participate in this study via the opt-out method on the dental clinic website. All teeth were include in this study, except third molars and teeth suitable for extraction (such as fractured tooth, impacted tooth, or tooth stump) at the initial visit. This study was conducted in accordance with the Declaration of Helsinki, the ethical committee of the Japanese Society of Periodontology approved this study in a matched collective (JSP2019001). And the ethical committee of the Japanese Society of Periodontology approved that the need for written consent would be waived via the opt-out method of the dental clinic website (JSP2019001). To maintain patient anonymity, personal information relating to the patients in this retrospective study was erased and participants’ names were replaced with ID numbers to analyze the data. The final analysis included 7584 teeth from 297 patients.

### Evaluation methods and items

Dental hygienists who worked at the dental office performed calibrated whole-mouth oral examinations. Periodontal pocket depth (PPD) was examined with periodontal probes (PCPUNC15; Hu-Friedy, Chicago, IL, USA) and the plaque control record was measured with a plaque disclosing solution (Satoh Dental Material, Tokyo, Japan) [19]. Oral examinations included PPD at 6 sites per tooth, bleeding on probing at 6 sites per tooth, the plaque control record at 4 sites per tooth [19], and the decayed, missing, and filled teeth (DMFT) index [20]. One calibrated dentist evaluated the tooth condition for tooth prosthesis and vital or non-vital teeth by using X-rays and electric pulp tests (Digitest; Parkell, NY, USA) [21–23]. Non-vital teeth were evaluated for the presence of root canal obturation (determined as > 2-mm dead space from the root apex) or there was no response by electric pulp tests referred from previous studies [16, 24].The dentist also counted tooth extraction in the clinic, and traumatisms, spontaneous avulsion teeth were excluded in this study. The general condition and smoking history of participants were also evaluated via medical interview; these data were self-reported by participants. Smoking history was categorized as the presence or absence of smoking experience up until the present. These oral examinations were evaluated at baseline (the initial visit) and at every visit with supportive periodontal therapy, and the clinical data of all teeth from oral examinations at the initial visit and latest visit were analyzed.

### Statistical analysis

Fisher’s test was used to compare the reasons for extraction between vital teeth and non-vital teeth. Kaplan–Meier survival plots were constructed and log-rank tests were performed for descriptive purposes between vital teeth and non-vital teeth. Cox hazards regression analysis was applied to determine risk factors (age, sex, smoking habits, diabetes mellitus, DMFT (DMFT was mean of the DMFT index which patient had subject’s tooth), PPD (PPD was evaluated the deepest pocket in 6 sites), molar, dental prosthesis, and non-vital pulp). Hazard ratios (HR) and 95% confidence intervals (CI) were used to assess the independent contribution of each identified risk factor. Statistical analyses were performed with EZR (Saitama Medical Center, Jichi Medical University, Saitama, Japan) [25], which is a graphical user interface for the open source statistical program “R” (The R Foundation for Statistical Computing, Vienna, Austria). More precisely, EZR is a modified version of R commander designed to add statistical functions frequently used in biostatistics. Statistical significance was set at *p* < 0.05.

## Results

### Study population

A total of 7584 teeth were analyzed in 297 patients. Table 1 shows the characteristics of teeth at baseline (mean age: 45 years old (standard deviation: 13 years), women: 72%). The mean follow-up time was 13.87 years (range 10.00–17.65 years, standard deviation 1.84 years); a total of 277 teeth were extracted during this period (There was no traumatism or spontaneous avulsion tooth during this survey period). Table 2 shows the reasons for extraction for all teeth (277 teeth were extracted during SPT). Fracture was the most common reason for extraction, accounting for 31% of cases (n = 87). Periodontal disease was also a common reason, accounting for 30% of cases (n = 82). The reasons for extraction differed significantly between vital teeth and non-vital teeth, according to Fisher’s test (Table 2, *p* < 0.001). The most common cause of extraction of a vital tooth was periodontal disease, whereas fracture was the most common cause for non-vital teeth. The percentage of extractions resulting from dental caries was twice as high for non-vital teeth as for vital teeth.

**Table 1:** The characteristics of patients and teeth at baseline

	Total n = 7584	Vital teeth n = 6347	Non-vital teeth n = 1237	*p* value
Year mean (sd)	45.28 (13.41)	44.06 (13.21)	51.54 (12.66)	< 0.001
Sex women n (%)	5492 (72.4)	4539 (71.5)	953 (77.0)	< 0.001
Smoking n (%)	1532 (20.2)	1300 (20.5)	232 (18.8)	0.175
Diabetes mellitus n (%)	202 (2.7)	169 (2.7)	33 (2.7)	1.000
DMFT mean (sd)	14.08 (6.48)	13.09 (6.28)	19.16 (4.92)	< 0.001
Molar n (%)	1987 (26.2)	1469 (23.1)	518 (41.9)	< 0.001
Prosthesis n (%)	1225 (16.2)	178 (2.8)	1047 (84.6)	< 0.001
PPD site mean (sd)	3.09 (1.27)	3.00 (1.22)	3.58 (1.44)	< 0.001
BOP site mean (sd)	1.04 (1.54)	0.96 (1.49)	1.44 (1.70)	< 0.001

Year mean = mean of the patient’s year which had subject’s tooth, Sex women = number of teeth which were refer women. Smoking = number of teeth which were refer smoking experience of the patients, Diabetes mellitus = number of teeth which refer diabetes mellitus patients, DMFT mean = mean of the DMFT index which patient had subject’s tooth, Molar = number of tooth which was molar, Prosthesis = number of tooth which was restored by crown or bridge as prosthesis, PPD site = mean of PPD site of the subject’s tooth, BOP site = mean of BOP site of the subject’s tooth

**Table 2:** Reasons for extraction of vital teeth vs non-vital teeth

Reason	Vital teeth	Non-vital teeth	Total
Periodontal disease	46 (47.9%)	36 (19.9%)	82
Dental caries	10 (10.4%)	51 (28.2%)	61
Fracture	27 (28.1%)	60 (33.1%)	87
Others^a^	13 (13.5%)	34 (18.8%)	47
Total	96	181	277

Fisher’s test *p* < 0.001

^a^“Others” are included trauma and unknown since other clinic extracted

### Survival probabilities for vital teeth and non-vital teeth

**Fig. 1:**
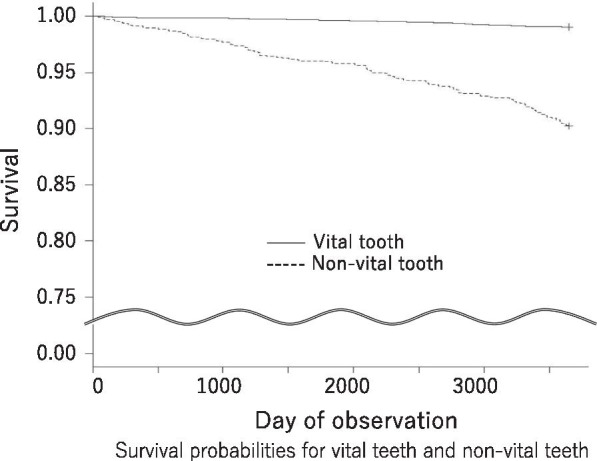
Survival probabilities for vital teeth and non-vital teeth

Figure 1 shows survival probabilities for vital teeth and non-vital teeth over a 10-year period. We observed a total of 7584 teeth and found an average annual extraction rate of 2.39% over an observation period of 10 years. The annual extraction rate for vital teeth was only 0.95% (n = 60) over a 10-year observation period, whereas the rate was 9.78% (n = 121) for non-vital teeth. This difference was statistically significant, according to the log-rank test (*p* < 0.001).

### Multiple classification analysis of risk factors for tooth loss

Crude associations between various baseline characteristics and tooth loss according to Cox hazards regression analysis are shown in Table 3. Non-vital pulp (i.e., non-vital tooth compared with vital tooth) had the strongest association with an increased tooth-loss rate, with a HR of 3.31 (95% CI 2.16–5.07; *p* < 0.001). Diabetes mellitus, smoking status, and PPD were also strongly associated with an increased tooth loss rate, with HRs over 2.0. All investigated factors except sex showed a significant association with tooth loss rates.

**Table 3:** Risk factors for tooth loss according to Cox hazards regression analysis

Variable	Hazard ratio	95% CI	*p* value
Year	1.064	1.052, 1.076	< 0.001
Sex (women)	0.780	0.585, 1.039	0.089
Smoking	2.180	1.613, 2.946	< 0.001
Diabetes mellitus	2.244	1.426, 3.533	< 0.001
DMFT	1.025	1.001, 1.050	0.040
PPD	2.169	1.671, 2.817	< 0.001
Molar	1.619	1.256, 2.089	< 0.001
Prosthesis	1.875	1.234, 2.849	0.003
Non-vital teeth	3.309	2.159, 5.070	< 0.001

## Discussion

The present study investigated the influence of various baseline parameters on tooth loss. A total of 7584 teeth in 297 patients were examined during a follow-up period of 10 to 17.65 years. We found that non-vital pulp was the most significant predictor of tooth loss in this study.

Many reports on the risk factors for tooth loss have not included an evaluation of non-vital pulp [7, 9, 11, 26–28]. A few studies have reported the relationship between tooth loss risk and non-vital teeth [17, 29, 30]. One case-controlled study reported that the odds ratio for loss of a root canal filled tooth was approximately 3.0 (compared with a non-root canal filled tooth) [29]. This finding does not conflict with those of the present study. Moreover, the same authors performed a matched cohort study and found that the HRs for tooth loss of root canal filled teeth versus non-root canal filled teeth were 7.4 (molars) and 1.8 (non-molars), respectively [30]. This finding is also similar to the results of the present study. However, these previous reports did not examine other important risk factors, such as clinical periodontal parameters, use of a prosthesis, and smoking habits. Therefore, it is difficult to compare our study with these previous studies.

There are many reports about the tooth survival rate after root canal treatment [31–33]. Fransson reported that the survival of root-filled teeth in Sweden was approximately 90% over a 5- to 6-year period [31], and Hong Kong was approximately 65% with root canal treatment tooth over a 10 year period [33]. In the present study, the survival probabilities of vital versus non-vital teeth showed very clear differences (Fig. 1). The survival rate of non-vital teeth continuously decreased whereas vital teeth maintained excellent survival. Therefore, it is strongly recommended that dental pulp extirpation should be limited to patients. In this study, various risk factors were evaluated. There were no significant differences in tooth loss between the sexes in our study, whereas other factors (age, smoking status, presence of diabetes mellitus, DMFT, PPD, molar location, and use of a prosthesis) showed statistically significant differences in Cox hazards regression analysis. Many studies have not found sex to be a risk factor for tooth loss [26–28, 34]. However, some studies have reported that male sex was a risk factor for tooth loss compared with female sex [13], and some reports showed female sex was risk factor in direct opposition [9, 14]. In our study, patients were in the supportive phase of periodontal therapy and they visited the clinic continually over a 10-year period without visit cessation. The subjects in previous studies were community residents who applied to participate in studies. Therefore, the included participants were totally different between our study and previous studies; the difference in outcomes with respect to sex may be related to these population differences.

We identified several risk factors that had a significant relationship with tooth loss. According to Cox hazards regression analysis, the HRs were 1.06 for age, 2.18 for smoking, 2.24 for diabetes mellitus, 1.03 for DMFT, 2.17 for PPD, 1.62 for molar tooth, and 1.88 for prosthesis use. These HR results were very similar to those of previous studies [7, 32, 34]. Therefore, our study was consistent with previous studies.

In contrast, the reasons for extraction in this study differed from those in previous studies [11, 13, 14, 17]. The reasons for extraction in the present study were tooth fracture, periodontal disease, dental caries, and others, in that order. In this study, teeth with poor prognosis were excluded (these teeth were extracted at initial treatment), and our tooth loss data were collected during the supportive phase of periodontal therapy. Therefore, differences in findings may have resulted from differences in the study populations. Moreover, the finding that fracture was the most common reason for extraction of non-vital teeth is very reasonable and confirmed the results of previous studies [17].

There are some limitations to the present study. First, we could not evaluate social factors, such as income, education, and occupation. In Japanese culture, a person’s socioeconomic and educational status can be very sensitive topics. However, we believe that socioeconomic and educational status were not associated with the decision to undergo the supportive phase of periodontal therapy because the participants all lived in the urban area of Hiroshima City and could therefore be expected to have similar lifestyles.

Second, evaluation of vital versus non-vital pulp was difficult in rare cases except in cases diagnosed with X-ray evaluation in clinical practice. However, we used electric pulp tests in the cases that were difficult to determine and attempted to be as objective as possible [21]. However, there are some evidences that cold pulp testing has better performance than electric test when assessing teeth vitality [35, 36]. Future studies, we should evaluate teeth vitality with cold test.

Third, this study was conducted with data from one dental clinic under SPT protocol. Selection bias was remained in our study. A multicenter study is necessary for generalization, and future prospective studies are needed to evaluate the risk ratio for tooth loss related to non-vital pulp.

## Conclusion

The present study showed that the teeth undergoing SPT were kept high survival rate by dental hygienists, and there was a significant relationship between non-vital pulp and tooth loss. Moreover, non-vital pulp had the most significant association with tooth loss among parameters evaluated (age, sex, smoking status, presence of diabetes mellitus, DMFT, PPD, molar location, and prosthesis use). Therefore, our findings suggest that it is very important to minimize dental pulp extirpation.

## Data Availability

The datasets used and analyzed in this study are available on reasonable request from the corresponding author.

## Abbreviations

BOP: Bleeding on probing
PPD: Periodontal pocket depth
DMFT: Decayed, missing, and filled teeth
SPT: Supportive periodontal therapy
HR: Hazard ratio
